# Morphological alterations of cultured human colorectal matched tumour and healthy organoids

**DOI:** 10.18632/oncotarget.24279

**Published:** 2018-01-19

**Authors:** Seyed Mohammad Hossein Kashfi, Sheema Almozyan, Nicholas Jinks, Bon-Kyoung Koo, Abdolrahman S. Nateri

**Affiliations:** ^1^ Cancer Genetics & Stem Cell Group, Cancer Biology, Division of Cancer and Stem Cells, School of Medicine, University of Nottingham, Nottingham, UK; ^2^ Wellcome Trust - Medical Research Council Stem Cell Institute, University of Cambridge, Cambridge, UK

**Keywords:** colorectal cancer, organoids, intestinal epithelium stem cell, transplantation, personalized medicine

## Abstract

Organoids have extensive applications in many fields ranging from modelling human development and disease, personalised medicine, drug screening, etc. Moreover, in the last few years, several studies have evaluated the capacity of organoids as transplantation sources for therapeutic approaches and regenerative medicine. Nevertheless, depending on the origin of the cells and anatomical complications, an organoid transplant may make tissue regeneration difficult. However, some essential aspects of organoids including the morphological alterations and the growth pattern of the matched tumour and their healthy derived organoids have received less attention. Therefore, the current work focused on culturing matched healthy and tumour organoids from the same patient with colorectal cancer (CRC) and assessed their timed growth and structural differences on a daily basis. The healthy organoids underwent proliferation and branching morphogenesis, while the tumour organoids did not follow the same pattern, and the majority of them developed cystic structures instead. However, the number and size of tumour organoids were different from one patient to another. The differential morphological changes of the healthy versus human colonic tumour organoids likely linked to distinct molecular and cellular events during each day. Thus, while their specific structural features provide valuable *in vitro* models to study various aspects of human intestinal/colon tissue homeostasis and CRC which avoid or replace the use of animals in research, this model may also hold a great promise for the transplantation and regenerative medicine applications.

## INTRODUCTION

Colorectal cancer (CRC) is the third most common malignancy in male and the second in the female worldwide with approximately 1.4 million new cases diagnosed in 2012 [1]. CRC ranks fourth in cancer-related mortality among all malignancies in the world. The majority of the CRC tumours arise from benign precursors known as the adenoma, which develops into invasive carcinoma [2]. Variations in treatment response and cancer progression patterns between individuals may be due to the heterogeneity of CRC tumours, which defined as interactions of the genetic background of tumour bulk cells with their environment [3]. In fact, the differences in genetic and epigenetic properties of each cell and their correlated tumours, construct the level of this heterogeneity as a whole tumour. At the cellular level, the morphological features of cancer cells can be demonstrated based on specific antibodies and immunofluorescence staining, which allow researchers to assess cellular events including cell proliferation, growth, differentiation, migration, and apoptosis within a tumour context. On the other hand, studying tumour diversity can be facilitated by the development of advanced models that closely resembles the *in vivo* tumour structure which may provide an alternative platform for the high-throughput studies and analysis. Therefore, in the last few years, a complex advanced 3-dimensional (3D) culture system known as organoids have been introduced as a model that mimics patients’ *in vivo* tissue in a variety of pathologic states [4–17].

The intestinal organoid as a 3D culture (which faithfully mimics the *in vivo* tissue from both intestinal crypts as well as single isolated *Lgr*5^+^ cells) was first established by Sato *et al.* [18, 19]. Several studies have since developed organoids from different mammalian organs [20–23]. Various essential exogenous growth factors and inhibitors are required to mimic the *in vivo* intestinal epithelial stem cell niche for the growth and expansion of organoids. These are including Wnt-3A, the Wnt agonist R-Spondin 1, the EGF and the BMP antagonist Noggin [18, 19, 24]. In recent years, many studies have conducted in culturing of the intestinal/colon organoids. To our knowledge, there are no systematic studies that communicate and compare the pattern of organoids growth/structure from normal and cancer-adjacent tissue derived from one patient or comparison with another patient.

Herein, using the human colorectal tumour and adjacent healthy derived organoids of patients with CRC, we aimed to observe and evaluate the morphological alterations and the growth pattern between the two derived organoids from the same patient *in vitro*. Tumour and adjacent healthy tissue samples of seventeen patients with colorectal cancer collected from Nottingham Health Sciences Biobank (NHSB) [Queens Medical Centre, the University of Nottingham]. Based on our observations, the growth pattern and morphology of the organoids derived from colorectal cancer tumour and adjacent healthy tissues from the same patient differed significantly. Hence, studying this model of *in vitro* 3D organoid culture can potentially reduce or replace the use of animals in research and may also be beneficial in minimising the complications associated with particular physiological conditions upon transplantation, through their healthy growth in patient's tissue.

## RESULTS

### Validation of conditioned media for organoid culture

To validate Wnt-3A, R-Spondin and Noggin conditioned medium, the TOP/FOP-flash luciferase reporter and Western blotting assays were carried out. The luciferase activity was significantly higher (P < 0.01) in cells treated with Wnt-3A together with R-Spondin (Supplementary Figure 1A), suggesting that these ligands can induce Wnt/β-catenin signalling *in vitro*. Furthermore, the western blotting analysis also confirmed the expression of the secreted Noggin protein in both monomer (32kD) and dimerised (64kD) forms [25] (Supplementary Figure 1B).

We had initiated our analysis by fixing and staining of organoids with the Phalloidin using of either whole mount sample or embedded paraffin sections [24]. Phalloidin functions by binding and stabilising filamentous actin (F-actin) (Supplementary Figure 2), but there was no any benefit to the live-imaging of an organoid with several focus levels forming a bright field stack, and by visualising the intensity variations of this stack using a phase contrast microscope. Thus, this study focused on the comparison of the two types of organoids using live organoids underlying the bright field and phase-contrast microscopy.

### Morphological changes of organoids derived from healthy colon epithelium

**Figure 1:**
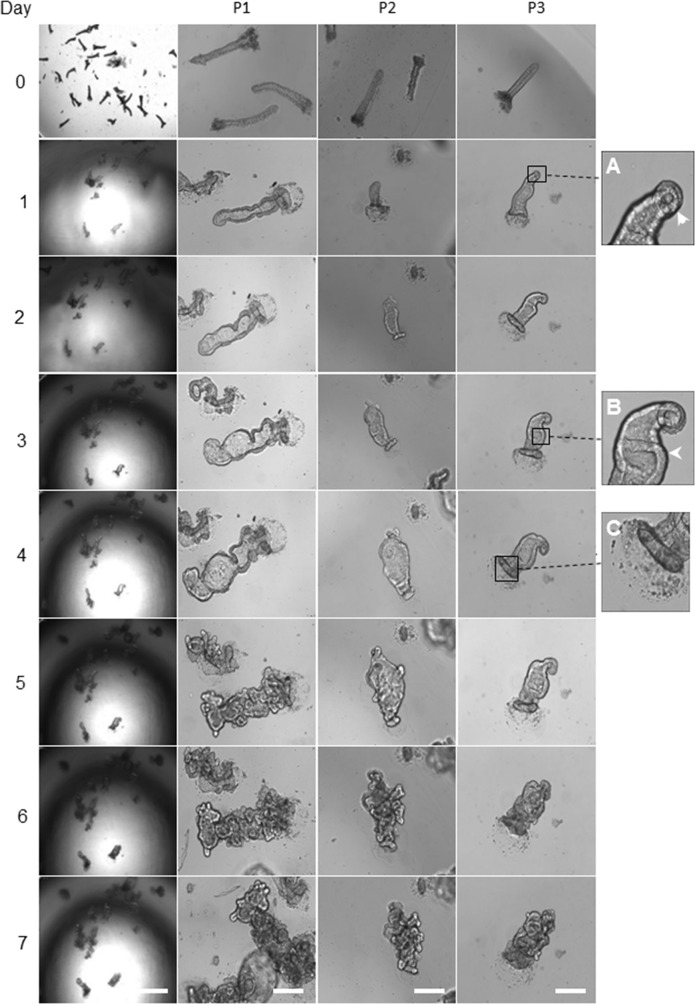
Representative images of morphology alteration in healthy colon organoids The left panel shows the gradual growth of organoids derived from the healthy human colon tissue adjacent to a tumour, day 0 to day 7. Scale bar, 250 μm. The remaining panels represent, the growth of healthy human colon-derived organoids from three different patients (P1, P2 and P3). Scale bar, 75 μm. The day zero crypts are representative images of cultured colonic crypts after seeding on the Matrigel, and it is not similar to the organoids showed for days 1 to 7. On day one, the crypt base region which enriched with stem cells started to grow **(A)**. From day 2 to day 3, the continuous gradual growth of organoid resulted in the colonoids structure with few budding domains **(B)**. On day 4, the top of the crypts sealed **(C)**. On days 4 to 6, the developing crypt was actively grown and differentiated during branching. The central area (lumen) in organoids contained the dead cells. On day 7, further, expansion created mature organoids, comprising numerous new regions of crypt-villus domains. Images in the first column captured by using a low magnification objective (1.25x) to focus mainly and to chase on the single/same type specimen for subsequent organoids growth. However, producing sharp and quality 3D images using lower than 10x-magnification is very difficult. We have therefore used 10x objectives for columns 2 to 4.

Upon seeding the isolated matched healthy colon crypts into the Matrigel, the organoid growth medium added, and the morphological changes imaged every 24 hours (Figure 1). On day one post-culturing, the crypts underwent circumnavigating (Figure 1A) then the elongated intact crypt grown into multi-cell spheroids (also known as colonoids) in the following days. From days 2 to 4, both crypts and colonoids possessed an enclosed central lumen and started budding into a new region with crypt-like structure (Figure 1B and 1C). The frequency of crypt budding increased rapidly during days 4 and 5 and the colonoids underwent excessive expansion resulted in an increase in the size of the organoids, leading to the formation of new multicellular budding and branched structures. The central region of the organoids contained dead cells resembling the intestinal lumen. During days 6-7, the mature organoids were either utilised for assays or further expanded in culture (Figure 1).

**Figure 2:**
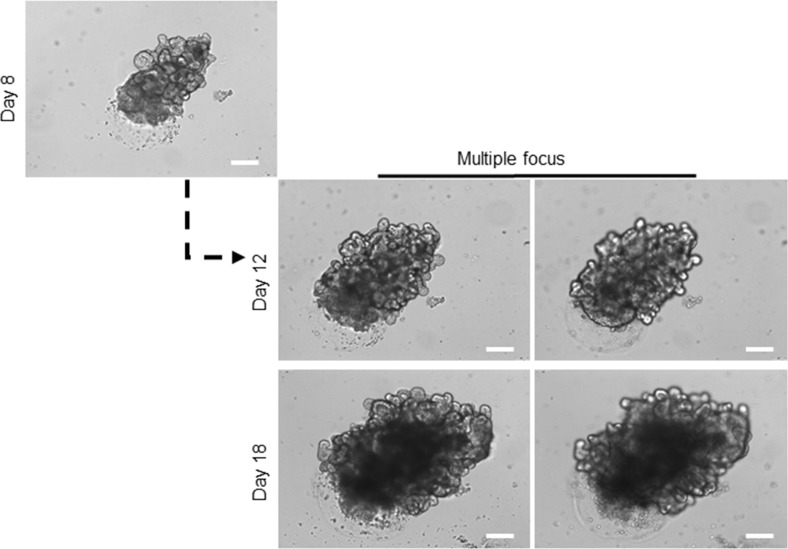
Long-term expansion of healthy organoids Growing the organoids over two weeks in culture steered the formation of a very large branched organoid compromising hundreds of crypt-villus domains. For details of branches in organoids, images captured with different focus lens. Scale bar, 75 μm. At this stage, the organoid can be broken apart, and single domain can be pooled in culture to form a new mature organoid, and it can be used to proceed the freezing process.

However, the continuous expansion of the organoids in culture for more than two weeks resulted in the formation of a very large mature organoid containing hundreds of crypt-villus like features, assembled both stem-cell and differentiated-cell compartments. Each of this crypt-villus like domains was capable of growing, budding, branching and formation of a new organoid, similar to the isolated crypt on the day zero (Figure 2). At this stage, these mature, healthy organoids can go under freezing methods for patient's own organoid biobank maintenance. Off-The-Shelf organoids will be needed if organoid transplantation enters a clinical phase.

In this study, we established organoids from seventeen CRC-patients and identified their growth status and structure alterations by comparison to a matched healthy organoids (Supplementary Table 1). However, under controlled conditions, it appeared that patients-derived organoids were similar to those organoids isolated from five individual patients in most cases.

### Morphological alterations of a colon cancer-derived organoids

**Figure 3:**
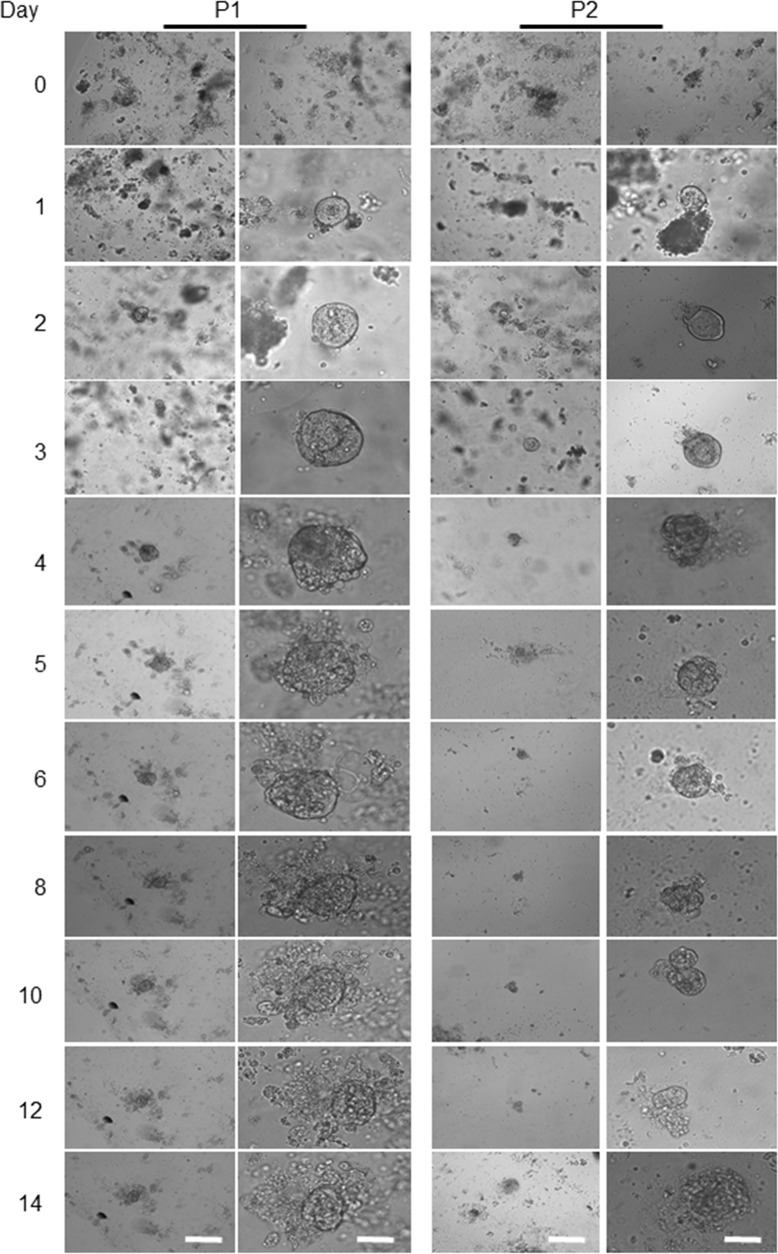
Representative images from patient-derived tumour organoids The four above panels are representing the human colon-derived organoids with different magnifications. Scale bars, 75 μm and 25 μm. The left two panels show the morphological alterations of the first patient’ (P1) a tumour derived organoid at different magnifications during days 4 to 14. The right two panels show another patient's derived tumour (P2) organoids grown and maintained for the same period. The lack of the intact crypt in tumour bulk is mainly due to the transformation of tumour cells as presented on day 0. Beside single cells, some other cells aggregated and formed clusters. Images in the first column, are captured by using a low magnification objective (1.25x) to focus mainly and to chase each specimen for subsequent organoids growth. However, producing sharp and quality 3D images using lower than 10x -magnification is very difficult. We have therefore used 10x objectives for columns 2 for each patient organoid.

**Figure 4:**
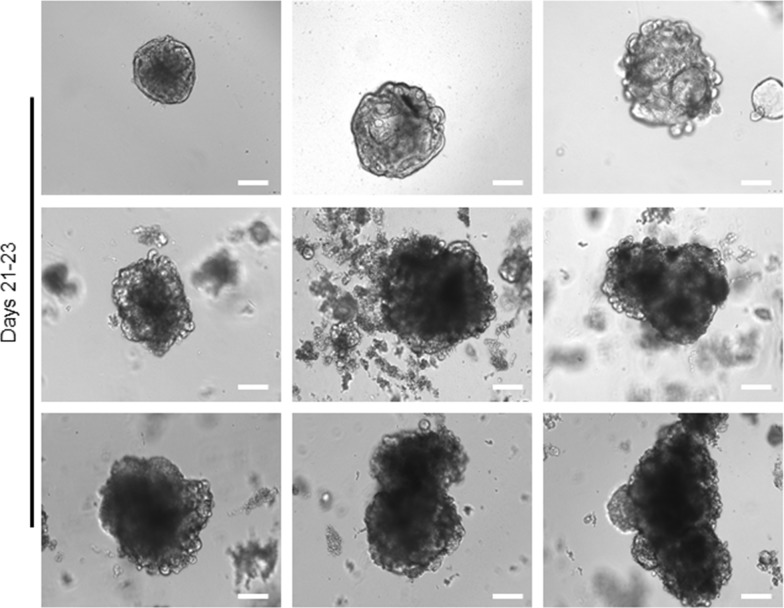
Long-term expansion of tumour organoids Representative bright-field images of single 14 day's tumour organoids from different patients that expanded in culture for further 7-10 days. Similar healthy organoids, the tumour organoids can develop for a long-term, while cannot actively differentiate and show heterogeneous phenotypes and morphology. Scale bar, 75 μm.

Unlike the adjacent healthy epithelium, no whole crypt-like structure observed in tumour samples at day zero, while some aggregated structures formed by a small population of cells (Figure 3). After seeding the tumour cells into Matrigel and adding organoid culture medium, a few cystic-like shape (representing the tumour derived organoids) with uneven borders appeared. In contrast to healthy organoids, these cystic-like structures failed to form budding or significant branching structures over a period of 4 to 14 days (Figure 3), possibly due to the deregulation of essential molecular and cellular signalling pathways required to maintain the colonic crypt proper function [26]. Further, to evaluate and confirm the degree of heterogeneity of the individual organoids in corresponding to the heterogeneity of CRC, we re-cultured the 14 days organoids for further 7-10 days (Figure 4). These data suggest that the additional genetic defects/changes or mechanisms intrinsic to cancer cells likely influence the organoid features.

**Figure 5:**
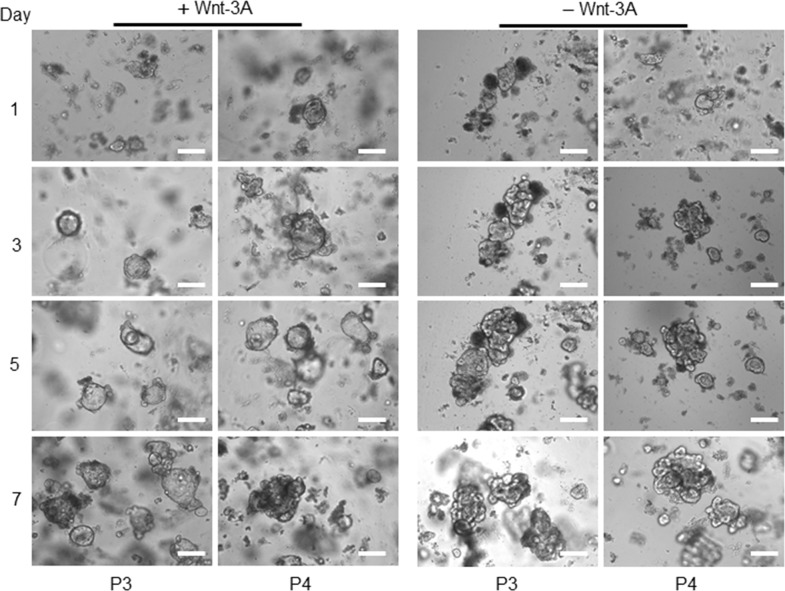
Tumour organoids growth in the presence and absent of Wnt-3A Representative bright-field images of tumour organoids grown in the presence or absence of conditioned Wnt-3A. Images captured from patients 3 and 4 (P3 and P4), scale bars, 75 μm.

**Figure 6:**
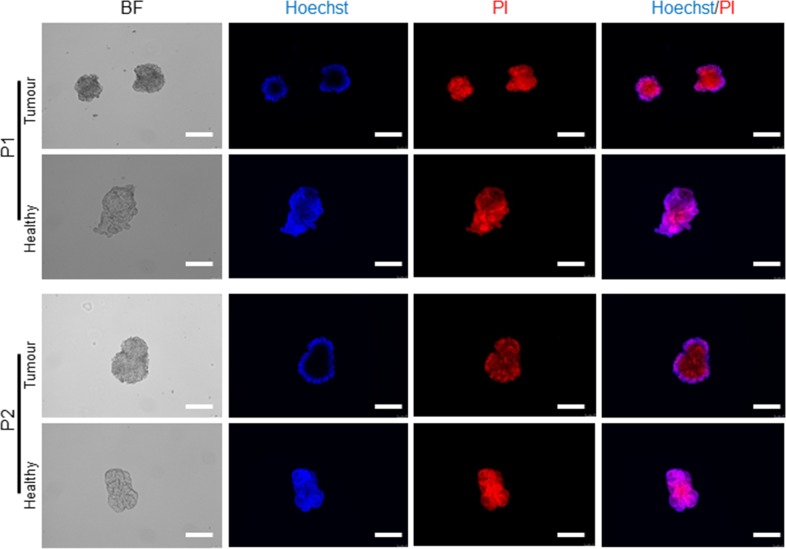
Live/dead staining in expanded tumour and healthy organoids Representative fluorescent images of organoids used for the analysis of cell death. Matrigel was disrupted mechanically and stained with a live/dead staining solution, contained Propidium iodide (PI) and Hoechst 33342 as outlined. Scale bars, 100 μm.

**Figure 7:**
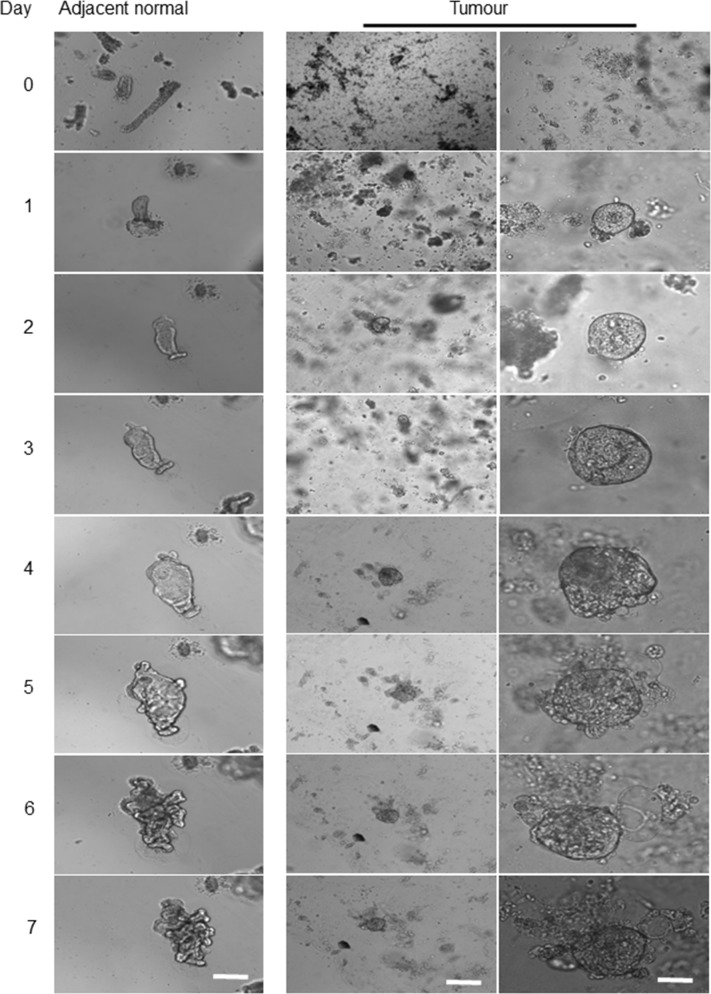
Comparison of morphological alterations in human colon matched a tumour and healthy organoids Differences in morphology and growth pattern of organoids derived from tumour/adjacent healthy tissue of patients with colon cancer within seven days implied the presence of different cellular and molecular process. Left panel represents the development of healthy colonic epithelium and middle panel indicates the growth pattern of a colonic tumour derived organoid. Scale bar, 75 μm. As the size of the tumour organoids was significantly smaller than their matched healthy samples on the same day, a higher magnification of a tumour derived organoid also showed (right panel, scale bar, 25 μm). In comparison to the healthy derived organoids, due to cell transformation and loss of differentiation activities, no intact crypt was detected in tumour mass after EDTA incubation. The growth pattern and size of a tumour derived organoids and their matched healthy tissue were significantly different with tumour organoids possess considerably a smaller size which slightly changed during the period.

To further investigate the extent of regenerative potential of tumour organoids upon on freeze-thawing and the abolition of canonical Wnt signalling, we re-cultured the frozen organoids from patients one and two respectively. Interestingly, the tumour organoids were capable of growing and resulted in an increase in the number of buds/lobes from their original cystic shape bodies in a growth medium without Wnt-3A (Figure 5). A recent finding supports this observation that the activated Wnt signalling pathway promotes the colon tumour organoid formation in Wnt Niche-independent signalling [27], and the abolition of canonical Wnt signalling improved their differentiation potential [28]. Furthermore, to investigate whether organoids with a disrupted and dark morphology regions in the bright-field microscope represent dead cells, we performed live/dead staining assay. Here, we used Hoechst 33342 dye, a popular cell-permeant nuclear counterstain for live-cell staining, and Propidium iodide (PI), which stain both viable cells and dead cells, on whole mount samples using both healthy and tumour organoids respectively (Figure 6). This data shows that the cell death responses and the number of dying cells in the healthy and tumour organoids are primarily different from each other. Also, a tumour crypt culture (days 4 to 14) possessed the cystic organoid structure with no cellular segregation and polarisation. The number of tumour cells capable of forming tumour organoids also varied from one patient to another. In some cases, very few tumour cells could grow into tumour organoid, while in other instances various tumour organoid structures were developed within a few days of seeding into the Matrigel. The observation indicates that not all the tumour cells of a bulk tumour are capable of generating tumour organoids. Moreover, the size of tumours-derived organoids was also different from the healthy-derived organoids (Figure 7). This data indicates that the structural features of a tumour derived organoids strongly mimic their tissue of origin.

## DISCUSSION

The genotype and phenotype of CRC tumours and their adjacent healthy tissues are significantly different *in vivo*. To provide a better insight into these differences a sophisticated *in vitro* model is required. Such a model should allow the propagation of primary tumours and the adjacent healthy tissues *in vitro* while maintaining the whole genome, transcriptome, and proteome of the original materials. Over the last few years, the intestinal/colonic organoids appeared as reliable 3D culture models to recapitulate the cellular and molecular features of the *in vivo* tissue growth.

Herein, we demonstrated the morphological changes of several tumours and their matched healthy tissues derived from CRC patients. The comparison of the morphology and growth pattern on daily bases indicated that both the tumours and adjacent healthy tissue obtained organoids possess fundamental difference in the phenotypical features, suggesting their molecular and genetic differences.

As previously reported, beyond 2-3 weeks, due to the consumption of growth factors and the subsequent induction of cell death, the old organoids display unhealthy state and activation of apoptotic processes. Therefore, these organoid cultures passaged, expanded and frozen. As a result, we have defined and described the organoid structures produced within 2-3 weeks in this study. It is of note that the tumour-derived organoids did not follow the similar growth pattern in all patients, likely due to differences in their age, genetic background, and medical history. Therefore, we cannot use organoids as a uniform tool across all patients; however, the improved methodology may highly increase the practicality of this model.

In the current study, the colon tumour and their adjacent healthy derived organoids were all grown in a similar shape and morphology presented in Figures 1–3. In this model, both a tumour and adjacent derived organoids remarkably reflected the morphology of *in vivo* counterparts, indicating their advanced capability as a model to study CRC progression. In line with our observations, previous studies have also confirmed the reliability of an intestinal tumour and adjacent healthy derived organoids in cancer studies [26, 27].

These studies together support the development of personalised medicine approaches for CRC treatment. In this regard, Van de Wetering and colleagues generated an organoid biobank from 20 patients diagnosed with colon cancer [26]. Through the genomic analysis of a tumour and matched healthy derived organoids, they reported that organoids showed the main subtypes that exist in CRC tumours. Furthermore, they designed organoid drug screening platform for therapeutic compounds before going into a clinical trial on patients, highlighting their potential in personalised medical treatments [26]. Similarly, Fujii *et al*. also established a library of organoids from 55 human CRC tumours and 41 matched healthy tissues. They confirmed that the growth of tumour organoids is not dependent on exogenous niche components in the prevailing culture, as the Wnt signalling pathway activated in the majority of CRC tumours. These findings are consistent with our current results that tumour organoids can grow without activated Wnt signalling (Figure 5). They also revealed that the organoids were histopathologically similar to the transplanted tumours and origin tumours [27]. Moreover, Cristobal *et al.* recent study on transcriptome and proteome of organoids derived from a tumour and adjacent healthy tissue of seven patients with colon cancer revealed that the organoids retained the original characteristics of CRC tumours where originated [29].

The application of organoids for regenerative and transplantation studies has also been addressed in recent works [15, 17, 30–34]. In one study organoids derived from Lgr5+ stem cells were transplanted into the damaged mouse colon, where after one month, functional crypts were formed at the damaged zone, highlighting the promising capacity of the organoids in the transplantation studies [17]. Furthermore, using the CRISPR-Cas9 genome editing system, Schwank *et al*. were able to correct the deletion at position 508 of cystic fibrosis transmembrane conductor receptor (CFTR) with homologous recombination on intestinal organoid from patients with CF, providing a potential application of the engineered organoid for gene therapies and transplantation approaches [15]. Recently, an engineered human intestinal tumour transplanted orthotopically into a mice model to study the colorectal tumour progression and metastasis has confirmed that the niche independency of distant tumours required the presence of at least four mutations [14].

On a broader scope, organoids hold a new hope in the area of regenerative medicine, particularly for organ transplantation purposes, where they can be used as a source of autologous tissue, avoiding the potential subsequent immune response and organ rejection upon transplantation [9]. However, an entirely successful transplantation could be difficult due to the limitations in the current delivery systems and the existing extracellular matrix (ECM) scaffolds such as Matrigel. Thus, more data are urgently needed on the reliability and validity of methods toward more reliable clinical or therapeutic practice. Such as the proper design of ECM and their compatibility with patients tissues [35], and the design of reliability studies in applying targeted delivery techniques like small molecules and expansion of organoids and graft survival [36]. Together, these studies have provided proof of concept for the regenerative and therapeutic utility of gut organoids and their promising future as a functional transplantable choice in different gastrointestinal (GI) diseases and injuries.

This study demonstrated for the first time, the morphological alterations in human colonic tumour and their matched healthy derived organoids, as all cell lineages can be fully differentiated, together with the above studies, represent a possible distinct molecular and cellular differences in their tissue of origin. Therefore, studying the organoid system will decisively contribute to the replacement and reduction of animal use. Furthermore, the growth of these organoids can be sensitised by changing culture conditions with growth factors/inhibitors, hence allowing investigation of multiple biological questions like cell cycle, apoptosis, cell signalling. These experiments are technically challenging to perform in a live animal, due to numerous inconveniences and physiologically variable parameters such as the route of administration and tissue distribution. Therefore becomes ethically costly as a large number of animals are required to have suitable statistical power for discovery science. Beyond these impacts, in principle, the development of such a large number of colonoids that phenotypically screened may provide valuable tools to enhance organoid transplant for regenerative medicine.

## MATERIALS AND METHODS

### Isolation and culturing of human colonic crypts

Tumour and adjacent healthy tissue samples of 17 patients with CRC collected from Nottingham Health Sciences Biobank (NHSB), Queens Medical Centre, University of Nottingham. Supplementary Table 1 shows the clinicopathological characteristics of patients enrolled in this study. A written informed consent was taken from each patient, and the local ethics committee approved the study protocol by the principles of the Helsinki Declaration. The tissue was washed with chilled PBS three times and cut into pieces of 5-10 mm. After incubation in 25 ml EDTA solution (1 mM EDTA in PBS) at 4°C for 40 minutes, the crypts were released from colon intestinal epithelium and subsequently collected. The crypts were re-suspended in Matrigel at a concentration of approximately 20 crypts/25 μL Matrigel. 25 μl crypt-containing Matrigel was carefully added to the centre of the well of a 48-well plate using chilled pipette tips. The plate was incubated at 37°C for 5 minutes to allow Matrigel to solidify. The Matrigel overlaid with 300-350 μL of complete organoid medium [18]. A 50 millilitre of complete medium supplemented with an advanced DMEM/F12, 50% of Wnt-, 20% of R-spondin-, 20% of Noggin- in-house conditioned media, 2% of B27 (x50, Invitrogen, 12587-010), 125 μl of 1.25 mM n-Acetyl Cysteine (Sigma, A9165), 250 μl of 10 mM Nicotinamide (Sigma, 98-92-0), 250 μl of EGF (100 μg/ml) (Sigma, E4127), 0.5 μM of A83-01 (TGF-β inhibitor, Tocris, 2939), 5 μl of 3 μM SB202190 (p38 inhibitor, Sigma, S7067) and 100 μg/ml of Primocin (InvivoGen, ant-pm-1).

### Preparation of noggin-conditioned medium

Noggin is post-translationally modified protein and mainly secreted as a disulphide-bonded homodimer [37, 38]. A secreted noggin protein binds and inactivates members of the transforming growth factor-β (TGF-β) superfamily signalling proteins, such as bone morphogenetic protein-4 (BMP4). Analysis of mouse mutants shows that this interaction is essential to modulate the activities of bone morphogenetic protein (BMP) during development [39]. In human, a reduction in the amount of secreted dimeric Noggin accounts for the human skeletal phenotypes [38, 40].

In this study, the HEK293T cells [American Type Culture Collection (ATCC)] transfected with 15 μg of Noggin plasmid using Polyethylenimine (PEI) in Opti-MEM (Thermo Fisher Scientific, 31985070). After transfection, the medium replaced with an advanced DMEM/F-12 (Invitrogen, 12634-028), supplemented with two mM L-glutamine, 100 U/ml Penicillin/Streptomycin (Pen/Strep) and ten mM HEPES (Invitrogen, 15630-122), and kept in the incubator for one week. After one week the medium was collected and centrifuged at 400 g for 4 minutes and filtered by 0.22 μM filters. Then aliquoted and stored at −80°C. Western blotting assay validated the presence of Noggin as a secreted protein.

### Western blotting

Western blotting performed as previously described by Li et al. [41]. An equal amount of total proteins denaturized, loaded into a 10% sodium dodecyl sulphate-polyacrylamide gradient gel (SDS-PAGE) for separation and then transferred overnight to polyvinylidene fluoride (PVDF) membranes. Membranes were blocked with 3% bovine serum albumin (BSA) in Tris-buffered saline and probed with purified mouse anti-Noggin antibody (BD Biosciences).

### Preparation of Wnt-3A- and R-Spondin 1-conditioned media

HEK293T expressing R-Spondin 1 (HA-Rspo1-Fc cell line) and HEK293T expressing an activated form of Wnt-3A (L-Wnt-3A cell line) received as a gift from Professor Hans Clevers (Hubrecht Institute, Netherlands). The Wnt-3A cell line was cultured in 20 ml of DMEM GlutaMAX supplemented with 10% FBS + Pen/Step and Zeocin (Thermo Fisher Scientific, R25001) (1.25 μg/ml). The flask was split into 6 × 175 cm^2^ flasks in growth medium with FBS, while the Zeocin added to only one container (for cell maintenance) and no Zeocin in the rest (5 × 175 cm^2^ flasks). Next, confluent cells were trypsinised, pooled in 600 ml growing medium (GlutaMAX) without Zeocin and plated in 30 × 150 cm^2^ dishes for one week. Then, after one-week incubation, the medium harvested. The collected medium containing the Wnt-3A were centrifuged at 400 g for 4 minutes and passed through 0.22 μM filter. Then the medium was aliquoted in and stored in 50 ml tubes at 4°C. The HEK 293T-HA-Rspo1-Fc cell line expressing R-Spondin 1 (R-spo1) grown and the medium collected as previously described [24]. The activity of the Wnt-3A and R-Spondin 1 (as a Wnt pathway agonist) was measured by TOP/FOP luciferase reporter assay.

### Luciferase reporter assay

To evaluate the transactivation activity of Wnt-3A and R-Spondin 1 conditioned medium, TOP-flash/FOP-flash dual luciferase reporter assay system (Promega #E1910) was exploited [42]. *Also, Renilla* luciferase (under SV40 constitutive active promoter) was used to normalise transfection efficiency. HEK293T cells were seeded in triplicate into a 6-well plate (3 × 105 cells/well) and kept in the incubator overnight. When the cells were 50-60% confluent, transfected with both *Renilla* luciferase vector (0.05 μg DNA/well) and, either TOP-flash or FOP-flash plasmids (0.2 μg DNA/well). The medium containing the transfection mixture was replaced with Wnt-3A + R-Spondin 1 conditioned media and left for 48 hrs. Then, the medium discarded and the cells were lysed and prepared for the bioluminescence reading according to the Kit instructions. The bioluminescence produced was detected by a Luminoskan Ascent Microplate Luminometer (Thermo Fisher Scientific, UK). Also, the measurement of the *Renilla* luciferase activity done by adding 50 μl/well of Stop & Glo Reagent to the same wells. The *Firefly* luciferase readings were normalised to *Renilla* luciferase readings and then to the activity of the control samples. The significance of differences between the mean and the median was determined using the Student's *t*-test and P < 0.05 (^*^) considered as significant.

### Organoids live/dead staining

Cell death in organoids assessed by live/dead staining and subsequent fluorescence confocal microscopy. Matrigel was disrupted mechanically with a pipette tip and organoids were then transferred to an Eppendorf tube. Live organoids stained with 50 μg/ml Hoechst 33342 (Sigma) and 50 μg/ml PI (Sigma) in the organoid medium. Organoids also fixed in 4% paraformaldehyde, washed with PBS, incubated with 100nM Alexa Fluor® 488 Phalloidin and incubated for 60 min in the dark. Then microscopy was performed using the Leica DMI3000 B fluorescent microscope at x10 or x40 magnification.

### Microscopy

The 3D organoids imaged at different zoom with objectives 1.25X, 5X, 10X and 40X using Leica DMI3000 B Manual Inverted Microscope. Representative bright-field images of organoids growths are across each day per group for three weeks in culture.

### Supporting information

Supplemental Information contains Supplementary Figures 1 and 2, Legends and Supplementary Table 1, can be found with this article online at *Oncotarget* website.

## SUPPLEMENTARY MATERIALS FIGURES AND TABLE





## Abbreviations

CRC: Colorectal Cancer
3D: 3-Dimensional
CFTR: Cystic fibrosis transmembrane conductor receptor
EGF: Epidermal growth factor
CRISPR: Clustered regularly interspaced short palindromic repeats
BMP: Bone morphogenetic protein
ECM: existing extracellular matrix
TGF-β: transforming growth factor-β.
