# Supplementary material for: Morphological alterations of cultured human colorectal matched tumour and healthy organoids
Source: Oncotarget. 2018 Jan 19;9(12):10572–84. doi: 10.18632/oncotarget.24279 (PMC5828197; doi:10.18632/oncotarget.24279)
Supplement: Supplementary file 1 [file oncotarget-09-10572-s001.pdf]

Morphological alterations of cultured human colorectal matched tumour and healthy organoids

SUPPLEMENTARY MATERIALS

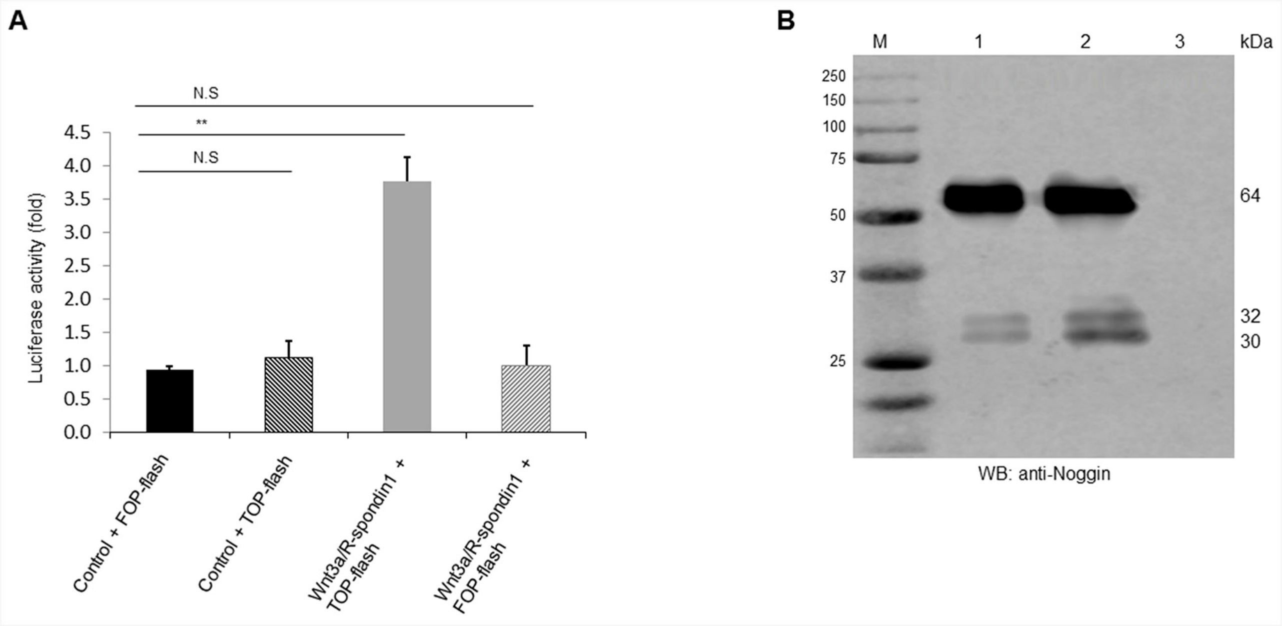

Supplementary Figure 1: (A) Luciferase reporter assay in HEK293T cells confirmed the Wnt-3A +R-Spondin function. The R-Spondin 1, as a Wnt agonist, induced the Wnt-3A activity. The luciferase activity was significantly increased in combination treatment of Wnt-3A +R-Spondin 1 $^{**}P < 0.01$. **(B)** Western blotting analysis of the secreted Noggin protein in HEK293 cells. The WB analysis of collected secreted Noggin protein revealed two bands with different sizes; monomer; 32kD and dimer, 64kD. Lane 1; represents the untransfected HEK293 cells, while lane 2; is a previously validated functional Noggin protein used as a positive control. Lane 3 shows cells transfected with Noggin plasmid.

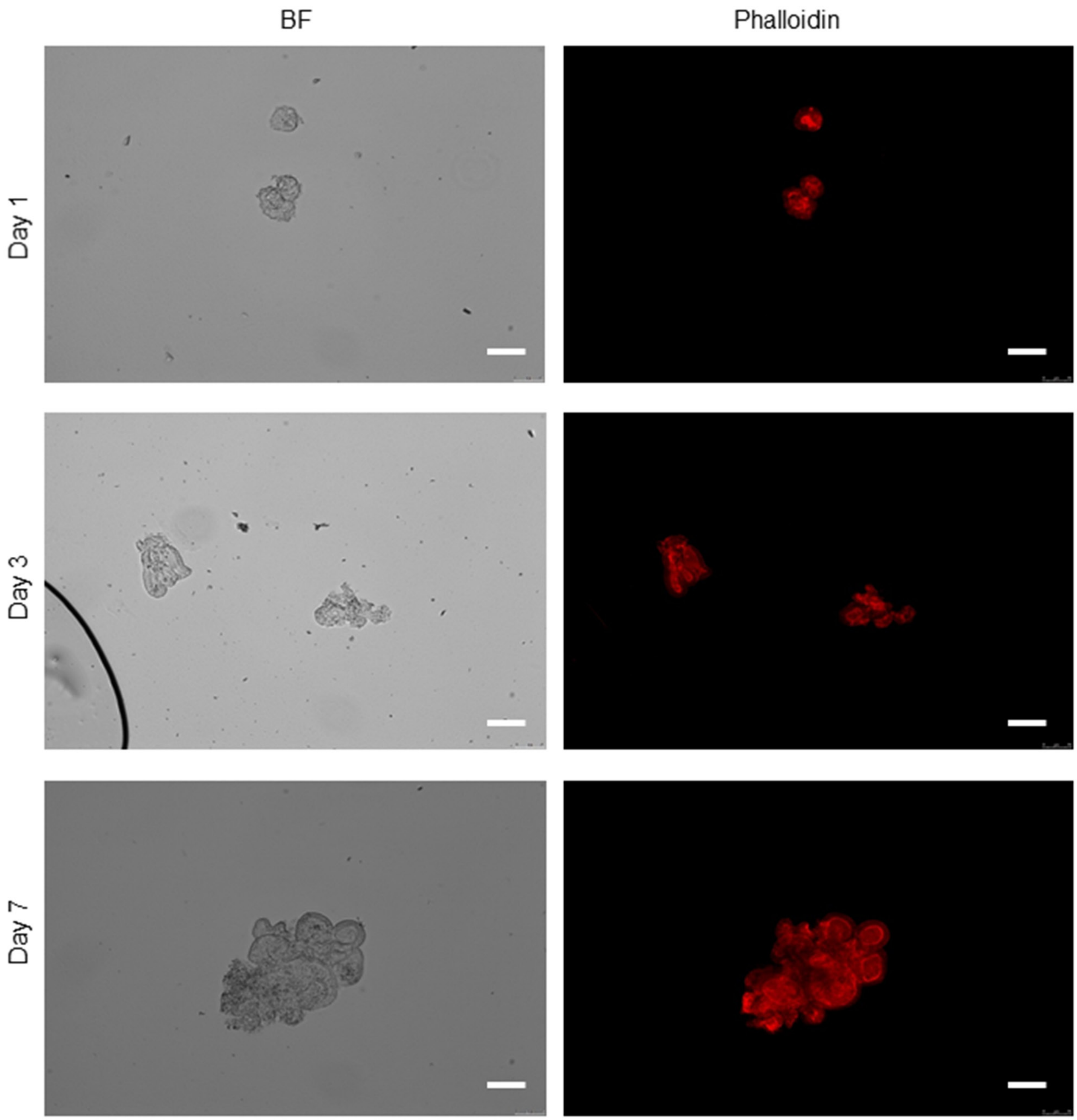

Supplementary Figure 2: Visualization of organoids morphology using either bright-field (BF) or fluorescent Phalloidin staining in single representative organoids. Scale bars, 100 µm.

Supplementary Table 1: The clinicopathological characteristics presentation of enrolled patients in the current study.

See Supplementary File 1
