# Supplementary material for: Morphological alterations of cultured human colorectal matched tumour and healthy organoids
Source: Oncotarget. 2018 Jan 19;9(12):10572–84. doi: 10.18632/oncotarget.24279 (PMC5828197; doi:10.18632/oncotarget.24279)
Supplement: Supplementary file 2 [file oncotarget-09-10572-s002.docx]

| **Patient No** | **Age** | **Gender** | **Diagnosis/history** | **Staging** |
| --- | --- | --- | --- | --- |
| 1 | 51 | M | Mid-rectal cancer with no interval liver or lung metastasis | T3 N0 M0 |
| 2 | 67 | M | Colonic carcinoma - poorly differentiated adenoma carcinoma | T3 N1 M0 |
| 3 | 69 | F | Colonic carcinoma - moderately differentiated adenoma carcinoma | T4 N2 M0 |
| 4 | 35 | F | Sigmoid colon polyp - tubular adenoma with focal high grade dysplasia | n/a |
| 5 | 79 | M | Colonic cancer - moderately differentiated adenocarcinoma | T2 N0 M0 |
| 6 | 62 | F | Colonic tumour - also got some lung nodules on the CT scan | T3 N2 M0 |
| 7 | 68 | F | Sigmoid colon cancer- moderately differentiated adenocarcinoma | T2 N0 M0 |
| 8 | 83 | M | Hepatic flexure cancer - moderately differentiated adenocarcinoma | T3 N1 M0 |
| 9 | 72 | F | Colonic carcinoma - moderately differentiated adenocarcinoma | T3 N3 M0 |
| 10 | 52 | M | Colonic cancer - moderately differentiated adenocarcinoma | T2 N0 M0 |
| 11 | 55 | M | Colonic cancer - moderately differentiated adenocarcinoma | T2 N0 M0 |
| 12 | 68 | M | Colon tumour with evidence of low GI cancer | T1 N0 M0 |
| 13 | 59 | M | Caecal carcinoma - poorly differentiated adenocarcinoma | T3 N0 M0 |
| 14 | 83 | M | Sigmoid cancer - Moderately differentiated adenocarcinoma | T3 N0 M0 |
| 15 | 62 | M | Rectal cancer - Moderately differentiated adenocarcinoma | T3 N1 M0 |
| 16 | 64 | F | Colon cancer - poorly differentiated adenocarcinoma | T3 N1 M0 |
| 17 | 69 | F | Colon cancer - moderately differentiated adenocarcinoma | T3 N1 M0 |
